# Assigning Clinical Significance and Symptom Severity Using the Zung Scales: Levels of Misclassification Arising from Confusion between Index and Raw Scores

**DOI:** 10.1155/2018/9250972

**Published:** 2018-01-21

**Authors:** Debra A. Dunstan, Ned Scott

**Affiliations:** School of Behavioural, Cognitive and Social Sciences, University of New England, Armidale, NSW 2351, Australia

## Abstract

**Background:**

The Zung Self-Rating Depression Scale (SDS) and Self-Rating Anxiety Scale (SAS) are two norm-referenced scales commonly used to identify the presence of depression and anxiety in clinical research. Unfortunately, several researchers have mistakenly applied index score criteria to raw scores when assigning clinical significance and symptom severity ratings. This study examined the extent of this problem.

**Method:**

102 papers published over the six-year period from 2010 to 2015 were used to establish two convenience samples of 60 usages of each Zung scale.

**Results:**

In those papers where cut-off scores were used (i.e., 45/60 for SDS and 40/60 for SAS), up to 51% of SDS and 45% of SAS papers involved the incorrect application of index score criteria to raw scores. Inconsistencies were also noted in the severity ranges and cut-off scores used.

**Conclusions:**

A large percentage of publications involving the Zung SDS and SAS scales are using incorrect criteria for the classification of clinically significant symptoms of depression and anxiety. The most common error—applying index score criteria to raw scores—produces a substantial elevation of the cut-off points for significance. Given the continuing usage of these scales, it is important that these inconsistencies be highlighted and resolved.

## 1. Introduction

Of the conditions contributing to the global disability burden of mental illness, anxiety and depression are the most prevalent disorders [1, 2]. However, while these are conceptually distinct constructs [3, 4], they present as highly comorbid conditions [5, 6]. Further, while an absence of positive affect is considered unique to depression, and other specific factors are unique to particular anxiety disorders (e.g., physiological arousal to posttraumatic stress disorder and panic disorder), the presence of a high level of general distress and negative affect is common to both types of disorder [7, 8]. For these reasons, researchers and clinicians often concurrently screen for the presence and severity of both disorders using self-report psychometric tools developed for this purpose.

Self-report measures of mental disorders may be criterion-referenced or norm-referenced. Criterion-referenced measures are used to make a diagnosis based on the endorsement of criteria listed in published diagnostic classification systems. Individuals are diagnosed with or without a disorder based upon the presence or absence of these criteria [9, 10]. In contrast to criterion-referenced measures, norm-referenced measures compare individuals' test results to those of an appropriate peer or normative group. These scales typically suggest score ranges linked to symptom severity descriptors and have a “clinically significant” total scale score cut-off point beyond which scores are considered indicative of the presence of a disorder (see Table 1).

The Zung Self-Rating Depression Scale (SDS) [11] is a commonly utilized norm-referenced scale. The SDS is a 20-item Likert scale covering symptoms that were identified in factor analytic studies of the syndrome of depression [11]. Items tap psychological and physiological symptoms and are rated by respondents according to how each applied to them within the past week, using a 4-point scale ranging from 1* (none, or a little of the time)* to 4* (most, or all of the time)*. The scale has a raw score range of 20 to 80 points. The raw score is then converted to an index score by dividing the raw score by the maximum score (80) and either expressing this as a decimal or multiplying by 100 to express it as a whole number with an index score range of 25 to 100. Index scores of 25 to 49 indicate nil depression, 50–59 indicate mild to moderate depression, 60–69 indicate moderate to severe depression, and scores over 70 indicate severe depression [12].

Zung [13] also devised a similar 20-item scale to screen for the presence of clinical anxiety: the Self-Rating Anxiety Scale (SAS). Items tap affective and somatic symptoms selected from the diagnostic criteria listed in the Diagnostic and Statistical Manual of Mental Orders (DSM II) current at the time [14]. The scoring structure and index score conversion is similar to that for the SDS. However, for the SAS, the situation regarding cut-off scores is less clear: Zung [6] noted in an early study that all “normal subjects” returned an SAS index score below 50, but later he set an index score of 45 (raw score = 36) as a cut-off point for clinically significant anxiety [15]. Moreover, score ranges for degrees of severity have not been published in the scientific literature.

Unfortunately, the literature reveals a number of discrepancies in the way the Zung scales have been used, reported, and interpreted. In particular, several researchers have mistakenly applied index score cut-offs to raw scores in assigning clinical significance and symptom severity ratings (e.g., [76, 118, 119]). In their Methods sections, these researchers describe the calculation of a total raw score and a “cut-off” score of 50 for morbidity. However, “50” is the* index* cut-off score set by Zung for the SDS, and this equates a raw score of 40. Using a raw score cut-off of 50 considerably reduces the proportion of cases classed as clinically significant. Another issue is that some researchers have applied severity range descriptors to the SAS when, as stated above, no such descriptors exist in the literature (e.g., [119]).

It is likely that errors in the scoring and interpretation of the Zung scales emanate from two sources that involve a failure to refer to the original publications. One is a reliance on the (erroneous) scale descriptions of other authors. The other is that some clinicians and researchers may have accessed scale information from sourcebooks of psychometric measures where distinctions between index and raw scores are imprecise. For example, both Fischer and Corcoran [120] and Schutte and Malouff [121] fail to clearly specify that recommended cut-off points are based on index and not raw scores.

This paper examines the extent to which these scales have been incorrectly interpreted in the literature. Given the scales' continued application, it is important that these inconsistencies in interpretation are highlighted and corrected.

## 2. Method

To investigate the extent to which the Zung scales are being wrongly applied, a search of the ProQuest full text database was conducted. Searches were done for each of the six calendar years from 2010 to 2015, using the terms “Zung Depression Scale,” “Zung Self-Rating Depression Scale,” “Zung Anxiety Acale,” “Zung Self-Rating Anxiety Scale,” and “Zung Self-Rating Scale.” Searches were limited to scholarly articles. For each calendar year, the results were examined in the order presented by the database search engine and, for both the SDS and the SAS, the first ten articles that used that scale to collect new data were selected to form a “convenience” sample indicative of recent use of these scales. Articles reporting on studies using both the SDS and the SAS were included, but theoretical articles and meta-analyses were not. In total, 102 articles were sourced and explored for misinterpretation of both scales. The disciplines covered in the articles were psychiatry (25%), psychology (9%), cardiology (6%), oncology (5%), neurology (5%), and gynecology (5%). The remaining 45% were other medical disciplines.

The results for each paper were recorded against a checklist. Examination initially focused on whether cut-off scores and severity ranges were applied. When this was done, the usage was coded according to the following categories:

1. *Consistent use of raw scores*: paper uses raw scores only with cut-off scores and/or severity ranges appropriately modified.
2. *Consistent use of index scores*: paper details conversion to index scores and uses index score cut-offs/severity ranges.
3. *Incorrect application*: index cut-off scores/severity ranges are specifically applied to raw scores.
4. *Unclear application*: paper uses index cut-off/severity ranges without mention of conversion from raw scores: however, there was no conclusive evidence that this was not done.
5. *Not utilized*: cut-off scores/severity ranges are not stated or used.

Notes were also taken where the cut-off and severity ranges applied were different from Zung's [12, 15] recommendations.

## 3. Results

Cut-offs for the presence of a disorder were applied in 45 of the 60 papers where the SDS was used and in 40 of the 60 where the SAS was used. For the SDS, index cut-offs were incorrectly applied to raw scores in 16 (35%) of these 45 papers, with a further 7 (16%) papers in which application was unclear. For the SAS, 8 (20%) of the 40 papers revealed incorrect application, with a further 10 (25%) being unclear (Table 2).

As shown in Table 3, the level at which cut-offs were set did not always accord with Zung's recommendations. In particular, alternative norms have been developed for use in Chinese populations with the cut-off for the SDS set at an index score of 53 (raw score, 42) and, for the SAS, at 50 (raw score, 40). Further, one of the SDS papers used the SAS cut-off (index 45, raw 36) and three of the SAS papers used the SDS cut-off (index 50, raw 40). Another three papers used the newly developed SDS cut-offs for a Chinese population but applied these to European samples. Finally, one of the SDS papers set a much higher cut-off index score of 60 (raw 48).

Severity ranges were utilized considerably less often. Specifically 23 of the 60 SDS papers included them but in 9 (39%) of these cases, index score ranges were incorrectly applied to raw scores, with a further 5 (22%) cases falling into the unclear category. Figures for the SAS followed a similar pattern despite the absence of any official ranges in the scientific literature. Twenty of the 60 SAS papers include such scales, with index score ranges being incorrectly applied to raw scores in 7 (35%) of these cases, with a further 7 (35%) falling into the unclear category (Table 4).

The most common severity range applied to the SAS is based on the recommended cut-off of 45 (index). In index score terms, severity ranges are 45–59 mild to moderate anxiety, 60–74 moderate to severe anxiety, and 75+ severe anxiety. Thirteen of the 20 SAS papers utilizing severity ranges employed the above. A further four used the SDS severity ranges, while two utilized different ranges altogether and the final paper merely specified descriptors without detailing the numerical criteria. The recommended SDS severity ranges were applied in all SDS papers but two, which instead used the “unofficial” SAS ranges detailed above.

## 4. Discussion

This study examined a sample of recent scientific publications for the application of raw scores, index scores, and symptom severity ranges when interpreting total scores on the Zung SDS and SAS. Although the findings were based on a “convenience” rather than a random sample of papers, they provide clear evidence of a significant problem in the application of Zung scales across the literature. On the basis of the papers examined here, confusion between raw and index scores means that when cut-offs are applied to indicate the presence/absence of disorder, they are applied incorrectly in 35–51% of cases for the SDS and 20–45% of cases for the SAS (depending on the proportion of unclear cases that involve incorrect application).

This incorrect application of index score cut-offs to raw scores substantially elevates the score required to be classified in the clinical range: in index terms, from 50 to 63 on the SDS and from 45 to 56 on the SAS. The potential impact on study findings does not need elaboration.

Quite apart from the issue of cut-off scores being incorrectly applied, the inconsistency introduced by the use of two distinct sets of scores to represent the same scale makes cross-study comparisons unnecessarily difficult. (Across the studies in our sample, raw scores were used approximately 40% of the time and index scores on 60% of occasions.) Given that the transformation to index scores achieves no purpose other than to decimalize the maximum score, the simplest solution might be to abolish the use of index scores altogether.

Additionally, some confusion exists between the two Zung scales with SDS cut-offs applied to the SAS and vice versa. The same applies to severity ranges for the two scales, if one accepts that an unofficial scale for the SAS has evolved in the literature. The scientific basis of this scale remains highly questionable.

The Zung scales continue to be widely used and potentially remain a valuable means of screening for the presence of anxiety and depression. However, if scale scores are to be reliably interpreted, it is a matter of some urgency that current confusion regarding scale cut-off and severity ranges is resolved and the application of these scales is standardized in future studies.

## Figures and Tables

**Table 1:** Clinical cut-off and severity ranges.

	SDS^1^		SAS^2^	
	Raw	Index	Raw	Index
Clinical cut-off	40	50	36	45
Severity range				
Mild-moderate	40–47	50–59		
Moderate-severe	48–55	60–69		
Severe	56+	70+		

*Note*. ^1^Zung (1974, pp. 176-177); ^2^Zung (1980, p. 18).

**Table 2:** Number of papers applying cut-offs correctly and incorrectly.

	SDS (*n* = 60)	SAS (*n* = 60)
Cut-offs not used	15	20
Consistent use: raw scores	9	8
Consistent use: index scores	13	14
Incorrect use	16	8
Unclear application	7	10

**Table 3:** Articles and evaluation of the use of the Zung Self-Rating Depression Scale (SDS) and Self-Rating Anxiety Scale (SAS).

Reference details	Discipline	SDS				SAS			
		Used?	Cut-off	Severity range	Notes	Used?	Cut-off	Severity range	Notes
*2010*									

Chagas et al., 2010 [16]	Neuroscience	✓	2	5					

Friebe et al., 2010 [17]	Psychiatry	✓	2	2					

Lande et al., 2010 [18]	Psychiatry	✓	2	2					

Saban et al., 2010 [19]	Psychiatry					✓	5	5	

Ohira, 2010 [20]	Cardiovascular	✓	1	5					

Lombardi et al., 2010 [21]	Immunology	✓	5	5					

Podlipný et al., 2010 [22]	Psychiatry	✓	2	2					

Ostojic et al., 2010 [23]	Rheumatology					✓	2	2	“SAS” severity ranges

Sonikian et al., 2010 [24]	Nephrology	✓	3	3					

Biggs et al., 2010 [25]	Psychiatry	✓	3	5					

Alimohammadi et al., 2010 [26]	Health					✓	5	5	

Oishi et al., 2010 [27]	Audiology	✓	1	5	Cut-off of 48 used (i.e., index of 60)				

Bitsika et al., 2010 [28]	Counselling					✓	1	5	

Sharpley et al., 2010 [29]	Oncology					✓	1	5	

Klemenc-Ketiš et al., 2010 [30]	Mental health					✓	1	5	Cut-off of 50 raw score

Tang et al., 2010 [31]	Mental health					✓	5	5	

Fernandes et al., 2010 [32]	Psychometrics					✓	1	1	Raw score ranges: up to 36 - no anxiety. 37–39 - possible anxiety: 40+ - high anxiety.

Wang et al., 2010 [33]	Urology					✓	2	5	cut-off 50 (Chinese)

Pascazio et al., 2010 [34]	Nephrology					✓	5	5	

Herbert et al., 2010 [35]	Psychology	✓	3	3					

*2011*									

Ide, 2011 [36]	Orthopedic	✓	1	1					

Lande et al., 2011 [37]	Psychiatry	✓	5	5					

Li et al., 2011 [38]	Psychology	✓	5	5	Short 10-item version of SDS used				

Huang et al., 2011 [39]	Cardiology	✓	4	4	Used index classifications with no indication of conversion				

Takayama et al., 2011 [40]	Dentistry	✓	1	1	Mentions index conversion but uses raw scores correctly				

Perugi et al., 2011 [41]	Psychiatry	✓	4	4	Used index scores with no indication of conversion	✓	4	4	Used index scores with no indication of conversion: cut-off of 50. SDS ranges

Ogawa et al., 2011 [42]	Gynecology	✓	5	5					

Uji et al., 2011 [43]	Psychotherapy	✓	5	5	Only used 7 statements: “affective subscale”				

Davidson et al., 2011 [44]	Psychology					✓	5	5	

Sharpley et al., 2011 [45]	Oncology	✓	1	5	SAS cut-off				

Wan et al., 2011 [46]	Psychiatry					✓	5	5	

Weigold and Robitschek, 2011 [47]	Psychiatry					✓	5	5	

Li et al., 2011 [48]	Opthalmology					✓	2	5	Chinese cut-off used (50 index)

Liao et al., 2011 [49]	Drugs					✓	5	5	

Nassiri et al., 2011 [50]	Env. science					✓	4	4	Divided into normal/low/moderate/high but no indication given of cut-offs or indexing

De Tommaso et al., 2011 [51]	Neurology					✓	5	5	

Chiaffarino et al., 2011 [52]	Gynecology					✓	1	1	Raw score ranges: <40, nonanxious; 40–60 anxious symptoms; >60 clinically significant anxiety

Richards et al., 2011 [53]	Psychology	✓	4	4	Uses Index scores: no mention of conversion	✓	4	4	Uses index scores with no mention of conversation. “SAS” ranges

*2012*									

Yu et al., 2012 [54]	Cardiology	✓	2	2		✓	2	2	“SAS” severity ranges

Lei et al., 2012 [55]	Psychiatry	✓	2	5	Chinese cut-offs used (53 index)	✓	2	5	Chinese cut-off used (50 index)

Mammadova et al., 2012 [56]	Psychiatry	✓	5	5	Calculating appropriate cut-off for different population				

Trento et al., 2012 [57]	Endocrinology	✓	3	3		✓	3	3	“SAS” severity ratings

Adogwa et al., 2012 [58]	Orthopaedics	✓	3	5					

Gao et al., 2012 [59]	Psychiatry					✓	1	5	Use Chinese norm cut-off of 40 (raw score)

Sawa et al., 2012 [60]	Psychiatry	✓	5	5					

Chang and Koh, 2012 [61]	Mental health	✓	5	5					

Sapranaviciute et al., 2012 [62]	Psychology	✓	5	5					

de Pasquale et al., 2012 [63]	General medicine	✓	3	5		✓	5	5	

Shen et al., 2012 [64]	Psychiatry					✓	2	5	Chinese cut-off: index 50

Liu et al., 2012 [65]	Gastroenterology					✓	2	5	Probably used Chinese cut-off

Huang et al., 2012 [66]	Gynecology					✓	2	5	Chinese cut-off: index 50

Li et al., 2012 [67]	Mental health					✓	5	5	

Tang et al., 2012 [68]	Gastroenterology					✓	5	5	

Campbell et al., 2012 [69]	Psychiatry	✓	5	5					

*2013*									

Adogwa et al., 2013 [70]	Spinal	✓	3	5	Other ranges used based on quartiles				

Balázs et al., 2013 [71]	Psychology					✓	4	4	“SAS” severity ranges

Li et al., 2014 [72]	Oncology	✓	1	5	Chinese cut-offs used (42 raw)				

Lowery et al., 2013 [73]	Psychiatry	✓	5	5	Brief instrument used. Score range indicated index scores (25 to 100) but only raw range given (1 to 4 Likert)	✓	5	5	Score range indicated index scores (25 to 100) but only raw range given (1 to 4 Likert)

Zhang et al., 2013 [74]	Urology	✓	2	5					

Guo et al., 2013 [75]	Oncology	✓	2	5	Chinese version used	✓	2	5	Chinese cut-off used

Nardelli et al., 2013 [76]	Neurology	✓	3	5					

Siennicki-Lantz et al., 2013 [77]	Geriatric	✓	2	2	SAS “severity rating” applied (i.e., mild depression 45–59)				

Liu et al., 2013 [78]	Immunology					✓	2	5	Chinese cut-off used

Deb, 2013 [79]	Pharmacology	✓	3	3		✓	3	3	SAS “severity ratings”

Wang et al., 2013 [80]	Psychiatry	✓	2	5	Chinese version used				

Khorvash et al., 2013 [81]	Neuroscience					✓	4	4	“SAS” severity ratings provided

Quintão et al., 2013 [82]	Psychology					✓	5	5	

Delibegovic and Sinanovic, 2013 [83]	Oncology	✓	4	4		✓	4	4	Uses SDS severity ratings

Klemenc-Ketiš and Peterlin, 2013 [84]	Psychiatry	✓	3	3					

Carli et al., 2013 [85]	Public health					✓	5	5	“A full description of assessment instruments and interventions was previously published”

Grandi et al., 2013 [86]	Gynecology	✓	5	5	“Higher scores indicating worst depressive symptoms.” Raw score range given (20 to 80)				

Luo et al., 2013 [87]	Geriatric					✓	5	5	

*2014*									

Akinsulore et al., 2014 [88]	Psychology	✓	2	2					

Banth and Sharma, 2014 [89]	Psychology	✓	3	3					

Bhatti et al., 2013 [90]	Spinal					✓	3	3	“SAS” severity ranges

Kaess et al., 2014 [91]	Psychiatry					✓	4	4	“SAS” severity ranges

Atteritano et al., 2014 [92]	Gynecology	✓	3	3					

Lee et al., 2014 [93]	Endocrinology	✓	5	5	“Severity score was calculated by formula conversion” and link to Zung article				

Vlachos et al., 2014 [94]	Gastroenterology	✓	3	5					

Ding et al., 2014 [95]	Psychiatry					✓	1	5	Chinese cut-off used (40 raw)

Trento et al., 2014 [96]	Endocrinology					✓	3	3	“SAS” severity ranges

Fernández‐Matarrubia et al., 2014 [97]	Neurology	✓	5	5		✓	5	5	

Feng et al., 2014 [98]	Public health	✓	4	5	Chinese cut-offs used (53 index)	✓	4	5	Chinese cut-offs used (50 Index)

Hou et al., 2014 [99]	Nephrology	✓	2	5	“Each scale consists of 40 items,”- incorrect description of scales provided. Chinese version used: non-Chinese cut-off	✓	2	5	“Each scale consists of 40 items,” incorrect description of scales provided. Chinese version used: Chinese cut-off

Khorvash et al., 2014 [100]	Neurology					✓	5	5	

Liu et al., 2014 [101]	Respiratory	✓	4	5	Chinese version used	✓	4	5	Chinese cut-off used

La Fianza et al., 2014 [102]	Radiology	✓	1	5		✓	1	5	`

*2015*									

Bobić et al., 2015 [103]	Psychiatry	✓	1	5	Chinese cut-offs used (42 raw)				

Chen et al., 2015 [104]	Orthopedic	✓	4	5	Chinese cut-offs used (53 index)	✓	4	5	Chinese cut-off used (50 index)

Jiang et al., 2015 [105]	Rheumatology	✓	5	5		✓	5	5	

Rus Makovec et al., 2015 [106]	Public health	✓	2	2		✓	2	2	SDS severity ranges used

Kourkoveli et al., 2015 [107]	Cardiac	✓	1	5					

Shi et al., 2015 [108]	Psychiatry					✓	2	5	Chinese cut-off used (50 index)

Stefanidou et al., 2015 [109]	Psychiatry	✓	3	5					

Pozzi et al., 2015 [110]	Psychiatry					✓	5	5	

Yuan et al., 2015 [111]	Neurology					✓	3	5	Chinese cut-off used (50 index)

Yin et al., 2015 [112]	Psychiatry	✓	3	3	SAS severity ranges quoted but Chinese cut-offs used (53 index)	✓	3	3	“SAS” severity ranges quoted but Chinese cut-off used (50)

Li et al., 2015 [113]	Spinal	✓	3	3	SDS standard indices applied to raw scores	✓	3	3	SDS severity ranges applied to raw scores

Hirao, 2015 [114]	Occupational therapy	✓	5	5	Mentioned the Japanese version				

Lou et al., 2015 [115]	Cardiac					✓	2	2	Chinese cut-off used (50 index): ranges: 50–59; 60–69; 70+

Yang et al., 2015 [116]	Psychiatry	✓	2	5	Mentioned the Chinese version but used standard index cut-off (50)				

Trento et al., 2015 [117]	Endocrinology					✓	3	3	“SAS” severity ranges

*Notes*. Zung analysis classifications; 1: consistent use of raw scores; 2: consistent use of index scores; 3: inconsistent application; 4: unclear whether consistent or not; 5: not utilized.

**Table 4:** Number of papers applying severity ranges correctly and incorrectly.

	SDS (*n* = 60)	SAS (*n* = 60)
Severity ranges not used	37	40
Consistent use: raw scores	2	2
Consistent use: index scores	7	4
Incorrect use	9	7
Unclear application	5	7
